# An Evaluation of a Train-the-Trainer Workshop for Social Service Workers to Develop Community-Based Family Interventions

**DOI:** 10.3389/fpubh.2017.00141

**Published:** 2017-06-30

**Authors:** Agnes Y. Lai, Sunita M. Stewart, Moses W. Mui, Alice Wan, Carol Yew, Tai Hing Lam, Sophia S. Chan

**Affiliations:** ^1^School of Public Health, The University of Hong Kong, Hong Kong, Hong Kong; ^2^Department of Psychiatry, University of Texas Southwestern Medical Center at Dallas, Dallas, TX, United States; ^3^The Hong Kong Council of Social Service, Hong Kong, Hong Kong; ^4^United Centre of Emotional Health and Positive Living, United Christian Nethersole Community Health Service, Hong Kong, Hong Kong; ^5^School of Nursing, The University of Hong Kong, Hong Kong, Hong Kong

**Keywords:** train-the-trainer, training program, positive psychology, Logic Model, family intervention

## Abstract

**Introduction:**

Evaluation studies on train-the-trainer workshops (TTTs) to develop family well-being interventions are limited in the literature. The Logic Model offers a framework to place some important concepts and tools of intervention science in the hands of frontline service providers. This paper reports on the evaluation of a TTT for a large community-based program to enhance family well-being in Hong Kong.

**Methods:**

The 2-day TTT introduced positive psychology themes (relevant to the programs that the trainees would deliver) and the Logic Model (which provides a framework to guide intervention development and evaluation) for social service workers to guide their community-based family interventions. The effectiveness of the TTT was examined by self-administered questionnaires that assessed trainees’ changes in learning (perceived knowledge, self-efficacy, attitude, and intention), trainees’ reactions to training content, knowledge sharing, and benefits to their service organizations before and after the training and then 6 months and 1 year later. Missing data were replaced by baseline values in an intention-to-treat analysis. Focus group interviews were conducted approximately 6 months after training.

**Results:**

Fifty-six trainees (79% women) joined the TTT. Forty-four and 31 trainees completed the 6-month and 1-year questionnaires, respectively. The trainees indicated that the workshop was informative and well organized. The TTT-enhanced trainees’ perceived knowledge, self-efficacy, and attitudes toward the application of the Logic Model and positive psychology constructs in program design. These changes were present with small to large effect size that persisted to the 1 year follow-up. The skills learned were used to develop 31 family interventions that were delivered to about 1,000 families. Qualitative feedback supported the quantitative results.

**Conclusion:**

This TTT offers a practical example of academic-community partnerships that promote capacity among community social service workers. Goals included sharing basic tools of intervention development and evaluation, and the TTT offered, therefore, the potential of learning skills that extended beyond the lifetime of a single program.

**Clinical trial registration:**

The research protocol was registered at the National Institutes of Health (identifier number: NCT01796275).

## Introduction

There is growing recognition of the importance of program theory in the development and evaluation of programs. However, reports of using theoretical models to develop programs in the social service sector are rare in the literature. The Logic Model offers an accessible framework that can be used to enhance capacity among frontline service workers with regard to the steps needed for program development and evaluation (1). This paper describes the development and evaluation of a train-the-trainer workshop (TTT) to develop (design and implement) community-based positive psychology family interventions. The workshop was delivered to social service workers (a combination of registered social workers and related-service workers) working in community agencies in Hong Kong.

Public health primary prevention requires effective, brief, and acceptable interventions to be delivered to large numbers of individuals in the community at low cost (2). Academic-community partnerships offer a synergy to develop large intervention programs (3). Academics bring the tools of science, whereas community service providers contribute insight regarding the needs of their constituents, and experience with the strategies that the community participants will find accessible and acceptable. As social service workers face constant changing needs, an even greater impact can be made if they can be provided with basic tools to adapt, develop, and evaluate new programs.

The Logic Model provides a simple road map for systematically developing interventions (4, 5). The model has been used as a planning and/or evaluation framework for a wide range of participants and settings, such as palliative care (6, 7); professional development programs for medical and health professionals (8, 9); programs for prevention of diabetes (10), HIV (11), breast cancer (12), and teenage pregnancy (13); research capacity in practice (14–16), primary care reform (17, 18); and community health promotions (19, 20). Although there has been increasing interest in the literature on planning and evaluation frameworks, the Logic Model has not been popularly used in the social service settings.

The train-the-trainer educational model has used experts to teach key stakeholders to deliver specific services (21) and has been broadly applied to workforce capacity building in health and social care settings (22–24). A TTT that teaches social service workers to use the Logic Model could more broadly enhance skills for the development and evaluation of programs. These skills could have considerable value in eventually maximizing the likelihood that programs being utilized in the community are based on local evidence. To our knowledge, there are no reports of TTTs designed to teach social service sector staff how to develop and evaluate simple programs to address needs in their community.

The FAMILY Project has been initiated and funded by The Hong Kong Jockey Club Charities Trust in collaboration with the School of Public Health of The University of Hong Kong (HKU-SPH). The aim of the FAMILY Project is to promote health, happiness, and harmony in Hong Kong (website: http://www.family.org.hk/) (25). In the first phase of the project, we conducted two TTTs for the Happy Family Kitchen Project (HFK) and the Enhancing Family Well-being Project (EFWB) to teach social service workers to develop community-based positive psychology family interventions (26, 27) leaving them with some freedom with regard to the specific content of their interventions. The second phase, the Happy Family Kitchen Project-II (HFK-II) aimed to increase public health impact by recruiting a larger sample of underprivileged families from different districts in Hong Kong and to respond to the expressed interest of frontline staff in the design and assessment of programs. The TTT presented in the current paper did not aim to teach a specific intervention, but rather to teach the general constructs of positive psychology in the context of family intervention development. The innovative component of our effort was that we provided frontline social service workers, who had little experience with research, with a framework for intervention development and evaluation in the form of the Logic Model.

Thus, in this paper, we examine the effectiveness of a training workshop for social service workers to develop community-based positive psychology family interventions. The primary outcomes were the changes in trainees’ knowledge, self-efficacy, attitude, and intention in the application of the general constructs of positive psychology and the Logic Model into the family interventions that they would develop after training. The secondary outcomes were trainees’ reactions to the training content, sharing of the knowledge, and benefits to their service organizations.

## Materials and Methods

### Participants

This was a single-group intervention study. Participants were staff assigned by the service organizations, schools, or government agencies, which participated in the HFK-II. Social service workers were eligible for the workshop if they were at least 18 years of age and were able to read Chinese and speak Cantonese. The participants were responsible for developing their own intervention programs in the community under the umbrella of the HFK-II interventions.

Ethical approval was granted by the Institutional Review Board (IRB) of The University of Hong Kong/Hospital Authority Hong Kong West Cluster (HKW IRB reference number: UW12-502) and the study was retrospectively registered at the National Institutes of Health (Identifier number: NCT01796275). Written informed consents were obtained from the trainees.

### The Intervention

#### The Needs of the Public and the Goals of the TTT

To plan for the HFK-II TTT, we established the context within which the service would be provided by examining recent surveys and statistics relevant to the Hong Kong public (28, 29) and feedback from our previous project (27). Surveys showed both perceived and actual increases in family conflict and violence in Hong Kong society (28). Long working hours, a stressful urban lifestyle, and a cultural tradition that does not emphasize the importance of direct acknowledgment and active engagement with family members posed barriers for positive intra-familial relationships (29). Feedback from the trainees who participated in the HFK TTT indicated that they needed more guidance and support in understanding the design, implementation, and evaluation of community interventions (26). Based on the above findings, the needs of social service sector, and the experience of conducting the previous TTTs (26, 27), we developed the training curriculum of the current TTT.

#### The Context of the TTT

Two TTTs were conducted for the HFK-II in May and September 2012. Each training workshop consisted of four 3-h sessions delivered over 2 days by a multidisciplinary research team (a clinical psychologist, an accredited dietician, registered social workers, and academic public health professionals with experience in developing intervention programs in the community). The training content covered the key components of the project, the five positive psychology themes for communication [“Joy” (30), “Gratitude” (31), “Flow” (32), “Savoring” (33) and “Listening” (34), nutrition, and the Logic Model (1, 5)]. Table 1 shows the curriculum of the training workshop.

**Table 1:** The curriculum of train-the-trainer workshop of the Happy Family Kitchen II Project.

Day 1	Day 2
**Session 1**	**Session 3**
Topic: Study introduction and theme-specific positive psychology session	Topic: Nutrition and research methods session
*Conducted by a program director and a clinical psychologist*	*Conducted by a dietician and academic public health professionals*
Goals: To introduce the key components of the projects (30 min) – Overall project aims – Conceptual framework – Family well-being	Goals: To introduce knowledge of healthy eating, choice of food, and the relationship between food and emotion (1 h)
To introduce specific positive psychology themes: “Joy” and “Praise and Gratitude” and their utilization for improving family communication with experiential activities (2 h and 30 min)	To explain the importance of healthy eating and demonstrate healthy and easy recipes (1 h)
	To introduce the key concept of the components of the project (1 h) – Evidence-based and evidence-generating research methods – Domains of process evaluations
**Session 2**	**Session 4**
Topic: Theme-specific positive psychology session	Topic: The Logic Model session
*Conducted by a clinical psychologist*	*Conducted by academic public health professionals and a program director*
Goals: To introduce specific positive psychology themes: “Flow,” “Savoring” and “Listening,” and their utilization for improving family communication with experiential activities (3 h)	Goals: To introduce the key concepts of Logic Model (1 h) – The components and outcome chain – The behavior indicator
	To demonstrate the application of the Logic Model and positive psychology themes in program design (1.5 h)
	To introduce study-related logistic arrangements including the evaluation method and measurement tools (30 min)

On the first day, the program director introduced the overall project aims and the conceptual framework of the family intervention in the HFK-II. Then, the clinical psychologist introduced the five positive psychology themes and the theme-related targeted behaviors through experiential activities. Each trainee has to develop his/her community-based positive psychology family intervention for his/her organization based on one of these five themes after training. The details of the five positive psychology themes have been reported in a separate paper (35).

On the second day, the dietician explained the importance of healthy eating and demonstrated how to make tasty and wholesome meals. Then, one of the academic investigators who was both a nurse and a public health professional introduced the Logic Model, demonstrated the application of the Logic Model in program development and involved the trainees in developing their family interventions based on this model.

A variety of in-class activities were conducted, including experiential games, role-play, small group discussions, and cooking demonstrations. Copies of the 117-page HFK-II training manual with practice guides for designing and implementing the positive psychology family interventions, and copies of the 61-page HFK-II cookbook with dietary information and healthy diet menu were distributed to the trainees.

#### Guiding the Design of the Community-Based Family Intervention

**Figure 1:**
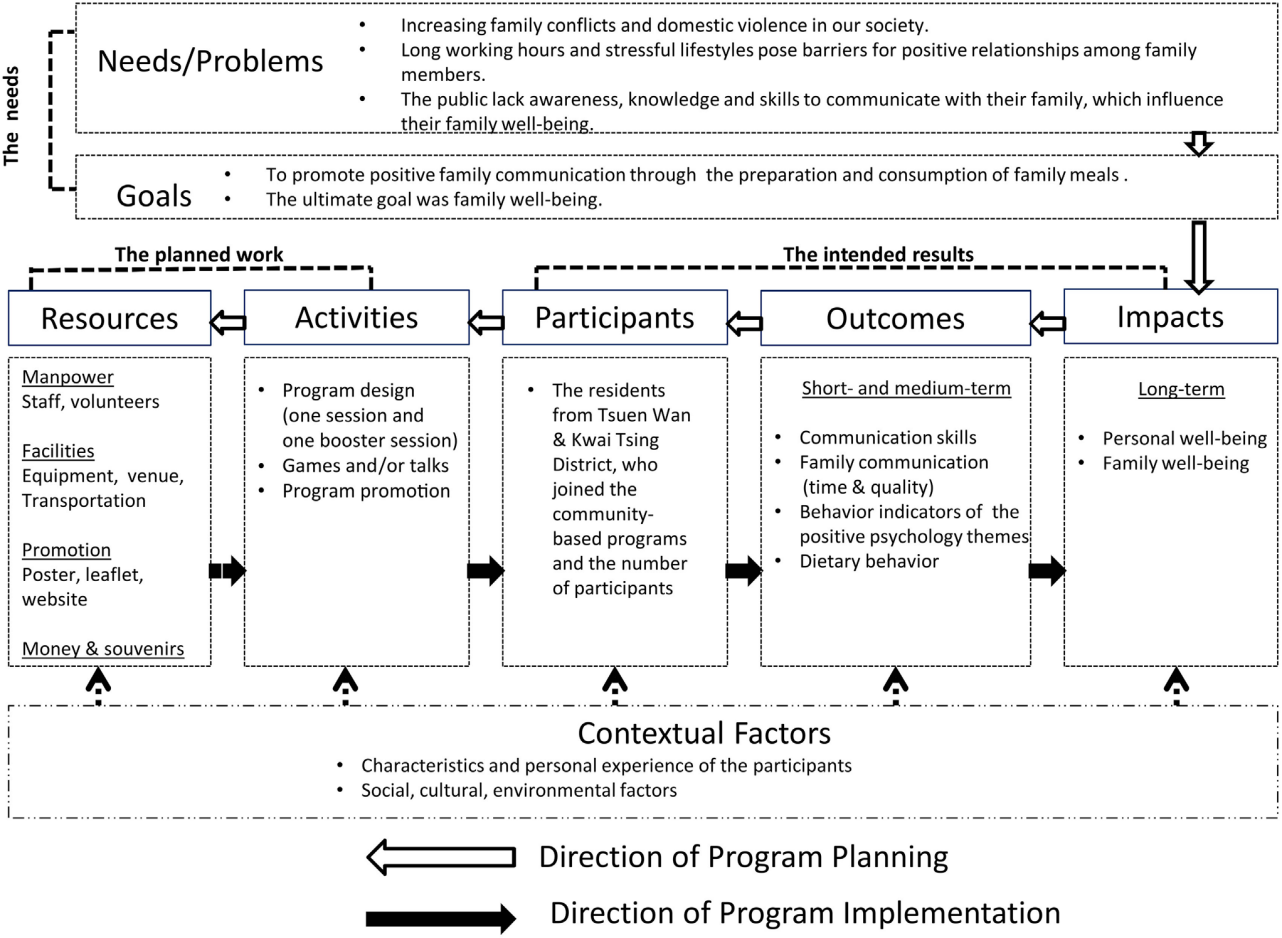
The Logic Model for the Happy Family Kitchen II Project.

During the training, we demonstrated how to use the Logic Model to guide the development of family interventions. Figure 1 shows the three major components guiding intervention design: the needs, the planned work, and the intended results.

##### The Needs

Guided by our findings in relation to the needs of families in Hong Kong, the interventions aimed to improve family well-being by strengthening family communication during the preparation and consumption of family meals.

##### The Planned Work

In providing a framework to develop their future projects, we first specified the intended outcome (positive family communication) as a path to the ultimate goal of family well-being. We directed the trainees to ask the following questions in sequential order: (i) “What outcomes/impacts would demonstrate that we have met our goals?”; (ii) “What kinds of behaviors do the participants need to change in order to achieve the targeted outcomes?”; (iii) “What knowledge or skills do the participants need to acquire before changing their behavior?”; (iv) “What types and number of participants do we need to recruit?”; (v) “What has to be in place for recruitment of participants?”; and (vi) “What resources are required for implementing recruitment and the behavior change program the trainees were to design?” Figure 1 shows the logistical sequential links between each step. We emphasized the importance of considering the contextual factors, which would be unique to each intervention. They included the participants’ characteristics (e.g., age and sex), personal experience, as well as social, cultural, and environmental factors. For example, a social service agency primarily serving physically disabled or mentally handicapped persons would anchor their project in different activities from those serving parents and young children. Different cultures and traditions needed to be respected during cooking demonstrations, such as no pork for Muslims and no meat for vegetarians.

##### The Intended Results

The Logic Model dictates that all outcomes/impacts should be “Specific,” “Measurable,” “Action-oriented,” “Realistic,” and “Timed” (36). These characteristics promote evaluation and eventually program improvement (37). Through the different family activities that would become part of each intervention, participants were expected to achieve positive short- and medium-term outcomes, specifically improvement in the quantity and quality of family communication, changes in dietary behavior, and changes in the behavioral indicators related to the positive psychology themes (Table S1 in Supplementary Material). We also expected that participants would achieve long-term goals, specifically improvement in personal well-being and family well-being (Figure 1).

### Data Collection

Both qualitative and quantitative evaluations were conducted. Self-administered questionnaires were used before and after the training and at 6-month and 1-year follow-up. Two semi-structured focus group interviews with the trainees were conducted after the implementation of the trainees’ first family intervention, at about 6 months after training, in November 2012 and March 2013.

The training workshop was evaluated on three dimensions: (i) changes in trainees’ learning; (ii) trainees’ reactions to training content; and (iii) knowledge sharing and benefits to trainees’ service organizations (long-term impacts).

### Measures

#### Changes in Learning

We assessed “changes in learning” by asking trainees to indicate the extent of their agreement with statements in relation to positive psychology and the Logic Model in the following categories: (i) cognitive learning (perceived knowledge) (three items; for example, “I know the key components of positive psychology” and “I have a basic understanding about how to apply the Logic Model in program planning.”); (ii) self-efficacy with regard to the information acquired (three items; for example, “I can master the techniques of positive psychology” and “I am capable of applying the Logic Model in program planning.”); (iii) attitudes toward the value of the concepts taught (six items; for example, “Positive psychology is effective at enhancing family well-being” and “The Logic Model can provide direction in program planning.”); and (iv) intention to apply the concepts acquired in their future programs (two items: “I intend to apply positive psychology in future activities” and “I intend to apply the Logic Model in program planning.”). Responses were made on a six-point Likert scale, ranging from “1 = strongly disagree” to “6 = strongly agree.” Cronbach’s Alpha of the subscales ranged from 0.89 to 0.92, showing good internal consistency.

#### Reactions to Training Content

We obtained trainees’ feedback on the training content and workshop by asking them to indicate the extent of their agreement with statements/their satisfaction in the following categories: (i) the performance of the speakers (four items; for example, “ The speakers presented clearly”); (ii) the applicability of the training content (six items; for example, “The training content was sufficient.”); and (iii) the overall satisfaction on the training workshop (10 items; for example, “The number of training sessions” and “Venue arrangement.”). Responses were made on a five-point Likert scale, ranging from “1 = strongly disagree/dissatisfied” to “5 = strongly agree/very good.”

#### Knowledge Sharing and Benefits to Trainees’ Service Organizations

We asked the trainees about whether in the past 6 months they had shared the knowledge they had acquired in relation to positive psychology constructs and the Logic Model 6 months and 1 year after attending the training workshop. Response options were “yes” and “no.” We also asked them to indicate changes in self-efficacy in relation to program design and policy development after the training. Responses on two items were made on a six-point Likert scale, ranging from “1 = no improvement at all” to “6 = improved a lot.” Scores from “1” to “3” and “4” to “6” were categorized as “no improvement” and “improved,” respectively.

Evaluation of the subsequent community-based family interventions that the trainees delivered has been reported in a separate paper (35).

### Data Analyses

Sample size calculation was based on change with a moderate effect size in perceived knowledge of the general concept of positive psychology at 1 year after training (26). Assuming a small attrition rate, 50 subjects were estimated as adequate for this study.

Analyses were conducted using SPSS version 20.0. All significance tests were two-sided with *p* < 0.05 indicating significance. Baseline values were used to replace the missing data of trainees who resigned from the participating organizations, who were lost to follow-up, or who declined to complete the questionnaire. Repeated measures analysis of variance and paired *t*-test were employed to compare data at four time points and between two time points, respectively. Data of the subgroup of trainees who had completed all assessments were also analyzed (per protocol analysis) to supplement the more conservative intention-to-treat analysis. The effect size (Cohen’s *d*) of the change in the outcomes was computed. This statistic reflects the magnitude of the difference, and unlike significance levels, it is not dependent on sample size. A positive effect size indicates an increase in the standardized mean score of the outcome, while a negative effect size indicates a decrease. Effect sizes of 0.2 to <0.5 have been described as small, 0.5 to <0.8 as medium, and 0.8 or above as large (38). All qualitative interviews were audio-taped and transcribed verbatim in Cantonese. Two project team members, one of whom attended the interviews, coded the transcripts. The transcripts were analyzed by thematic content analysis, following the guidelines recommended by Morse and Field (39).

## Results

**Figure 2:**
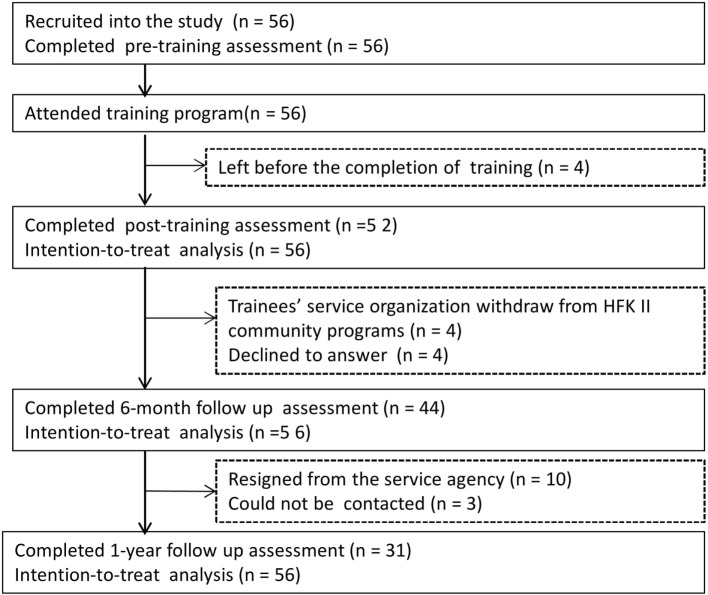
CONSORT diagram for the train-the-trainer workshop.

Fifty-six trainees, including 41 registered social workers, 13 related-service workers, and two teachers, attended the training workshops and were included in this study (Table 1). Four trainees attended the workshops, but left before completing the post-training assessment. At 6-month follow-up, four trainees did not respond because their corresponding service organizations had withdrawn from the HFK-II prior to the start of the intervention programs, and four trainees declined to complete the questionnaire. At 1-year follow-up, 10 trainees had resigned from their service organizations and three trainees could not be contacted. Fifty-six completed questionnaires at pre-training, 52 completed immediately after training, and 44 and 31 trainees completed at 6-month and 1-year follow-up, respectively, and all these questionnaires were thus collected (Figure 2). Thirteen trainees participated in the focus group interviews.

Table 2 shows the characteristics of trainees. The demographic characteristics of trainees who completed the 1-year questionnaire and those who did not and of trainees who joined the focus groups and those who did not were not different (Table S2 in Supplementary Material).

**Table 2:** Demographic characteristic of all trainees, those who completed the 1-year follow-up and those who participated in the focus group interviews.

	All (*n* = 56)	Completed the 1-year follow-up (*n* = 31)	Participated in the focus group interviews (*n* = 13)
	
	Number (%)		
Age group, years			
18–24	4 (7)	1 (3)	2 (15)
25–34	34 (61)	17 (55)	8 (62)
35–44	15 (27)	10 (32)	2 (15)
≥45	3 (5)	3 (10)	1 (8)
Female	44 (79)	24 (77)	9 (69)
Tertiary degree or above	41 (73)	22 (71)	9 (69)
Occupation			
Registered social worker	41 (73)	25 (81)	13 (100)
Service worker	13 (23)	5 (16)	0 (0)
Teacher	2 (4)	1 (3)	0 (0)
Social service experience			
Less than 5 years	19 (34)	7 (22)	6 (46)
5–9 years	18 (32)	12 (39)	1 (8)
≥10 years	19 (34)	12 (39)	6 (46)
Service targets			
Family	34 (61)	22 (71)	6 (46)
Children	28 (50)	13 (42)	7 (54)
Teenagers	23 (41)	12 (39)	6 (46)

### Changes in Learning

Table 3 shows significant increases in perceived knowledge, self-efficacy, and attitude in relation to positive psychology with moderate to large effect sizes immediately after training (Cohen’s *d*: 0.67–1.43). These effects were sustained at 6 months (Cohen’s *d*: 0.45–1.12) and 1 year (Cohen’s *d*: 0.39–0.83). The intention to apply positive psychology in interventions significantly increased with a small effect size immediately after training (Cohen’s *d*: 0.48), but was not sustained at 6-month and 1-year follow-up.

**Table 3:** Trainees’ perceived knowledge, self-efficacy, attitude, and intention to apply the concepts of positive psychology and the Logic Model in community interventions over time: intention-to-treat analysis.

*n* = 56	Pre-training	Immediately following training	6 months	1 year	Difference between		
					Pre-training and immediately following training	Pre-training and 6 months	Pre-training and 1 year

Positive psychology	Mean score ± SD				Cohen’s *d*^a^/*p*-value		
Perceived knowledge of the general concept of positive psychology^#^	3.2 ± 1.1	4.7 ± 0.8	4.3 ± 1.1	4.0 ± 1.3	1.28/<0.001***	0.86/<0.001***	0.68/<0.001***
Self-efficacy in relation to using positive psychology constructs to design interventions^#^	2.8 ± 1.0	4.3 ± 0.9	4.0 ± 1.1	3.7 ± 1.2	1.43/<0.001***	1.12/<0.001***	0.83/<0.001***
Attitude toward the practice of positive psychology^#^	4.3 ± 1.0	4.9 ± 0.8	4.7 ± 0.9	4.6 ± 0.9	0.67/<0.001***	0.45/<0.01**	0.39/<0.01**
Intention to apply positive psychology in interventions^#^	4.6 ± 1.0	5.0 ± 0.8	4.7 ± 0.9	4.7 ± 0.9	0.48/<0.01**	0.05/0.73	0.02/0.86

**The Logic Model**							
Perceived knowledge of the general concept of the Logic Model^#^	3.0 ± 1.1	4.4 ± 0.9	4.1 ± 1.0	3.6 ± 1.1	1.23/<0.001***	0.99/<0.001***	0.60/<0.001***
Self-efficacy in relation to using the Logic Model to design interventions^#^	3.6 ± 0.9	4.4 ± 0.9	4.2 ± 0.9	3.8 ± 0.8	0.86/<0.001***	0.57/<0.001***	0.29/<0.05*
Attitude toward the practice of the Logic Model^#^	3.6 ± 1.0	4.5 ± 0.8	4.3 ± 0.9	3.9 ± 0.9	0.80/<0.001***	0.65/<0.001***	0.36/<0.05*
Intention to apply the Logic Model in interventions^#^	3.7 ± 0.9	4.5 ± 0.9	4.2 ± 0.8	3.9 ± 0.8	0.83/<0.001***	0.50/<0.01**	0.22/0.11

*Number of questions on positive psychology: perceived knowledge (2 items), self-efficacy (2 items), attitude toward the practice (3 items), and intention to apply in practice (1 item)*.

*Number of questions on the Logic Model: perceived knowledge (1 item), self-efficacy (1 item), attitude toward the practice (3 items), and intention to apply in practice (1 item)*.

*Six-point Likert scale: 1 = strongly disagree; 2 = disagree; 3 = slightly disagree; 4 = slightly agree; 5 = agree; 6 = strongly agree*.

*Repeated measures analysis of variance and paired T-test comparing the mean at four time points and between two time points, respectively*.

*Difference at four time points: ^#^all p-value < 0.001*.

Difference between two time points: ***p value < 0.001, **p value < 0.01, *p value < 0.05.

*^a^Effect size (Cohen’s *d*): small = 0.20, medium = 0.50, and large = 0.80*.

Trainees showed significant increases with large effect sizes in perceived knowledge, self-efficacy, and attitude in relation to the Logic Model immediately after training (Cohen’s *d*: 0.80–1.23). These effects were sustained at 6 months (Cohen’s *d*: 0.57–0.99) and at 1 year (Cohen’s *d*: 0.36–0.60). The intention to apply the Logic Model in program design significantly increased immediately after training with moderate to large effect size (Cohen’s *d*: 0.83) and was sustained at 6 months (Cohen’s *d*: 0.50). Per protocol analyses on the smaller group of trainees who completed the 1-year follow-up showed similar findings (Table S3 in Supplementary Material).

### Reactions to Training Content

Immediately following training, over 90% of the trainees agreed or strongly agreed that the speakers presented clearly and that the training content was applicable and appropriate. Over 80% of the trainees rated the overall evaluation of the workshop as “good” or “very good.”

At focus group interviews, the trainees indicated that the training was well organized, practical, and comprehensive. The training enriched their knowledge on the general concept of positive psychology and the Logic Model, and enhanced their competence and confidence on using positive psychology and the Logic Model in their family interventions.

(The training related to) positive psychology was good …. We learned new ideas, and reviewed what we had learned in the past. It was so practical and comprehensive that we could clearly understand the content …. It gave us more confidence …. (Project designer and implementer, woman, aged 33 years)

I feel that the content of the training workshop gave me a clear direction (in program design). Information (about the Logic Model) inspired me and also helped a lot in designing in-service staff training. My colleagues and I also discussed the utilization of the Logic Model in various scenarios. (Project designer and implementer, man, aged 28 years)

### Knowledge Sharing and Benefits to Trainees’ Service Organizations

The trainees successfully developed (designed and implemented) 31 family intervention programs for almost 1,000 families at about 3 months after the TTT. The details of the trainees’ developed programs had been reported in a separate paper (35).

At 1-year follow-up, 67 and 36% of trainees reported that they shared the knowledge and techniques that they had learned about the general concept of positive psychology and the Logic Model with their colleagues, respectively. Ninety-four percent of the trainees reported that there were improvements in designing and implementing community and/or family activities programs, and 74% of the trainees reported that their organizational policies on the development of family programs were positively influenced as a result of their acquired new knowledge.

At the focus group interview, trainees reported that the training manual was useful, and the information they received had been applied to other services in their organizations.

In the past few years, my center wanted to promote the application of positive psychology in our centers (but we had no idea how to incorporate this into our programs). The tools (training manual) we received are good and we can use them in our future programs …. (Project designer and implementer, woman, aged 33 years).

I have been applying the concepts of positive psychology …. I feel that it was worthwhile to participate in the train-the-trainer workshop …. (The workshop) was practical and useful, and also useful to other services (offered in our agency) …. I applied (the concepts I had learned) to a stress management group. (Project designer and implementer, man, aged 30 years)

## Discussion

The trainees indicated a high level of satisfaction with the workshop. The TTT enhanced trainees’ competence (perceived knowledge and self-efficacy) and attitudes toward using positive psychology constructs and the Logic Model to develop family interventions. The findings ranged from small to large effect and were sustained to 1 year following the workshop. The current TTT adds to the literature as follows: (i) we provide a practical example of development (including design and implementation) and evaluation of a training workshop and (ii) we provide a template for applying the Logic Model in developing community-based family intervention programs.

Our findings are consistent with those from the TTTs of our previous projects (26, 27): both the HFK TTT and the EFWB TTT enhanced trainees’ competence and attitude in relation to using positive psychology to develop community-based family interventions. The effect size was the greatest in the current study (Cohen’s *d* ranged from 0.39 to 1.43), compared with those of the HFK TTT (Cohen’s *d* ranged from 0.37 to 1.22) and the EFWB TTT (Cohen’s *d* ranged from 0.24 to 1.06) (26, 27). The greater effect size was possibly because the current workshop was the third one we designed in the FAMILY Project to teach social service sector workers to apply positive psychology constructs in family interventions. The experience gained from the previous TTTs informed the selection of positive psychology themes and refinement of the training content, thereby enhancing the current TTT’s applicability and acceptability.

Implementation science is a rapidly growing discipline. The current TTT introduced the Logic Model, a simple tool with a clear structure and pictorial description, intended to be useful over the lifespan of a program from design to implementation and evaluation. The Logic Model helped the trainees to articulate each component of the entire program and consider the contextual factors of the participants. It facilitated the identification and collection of data for detecting behavior change and promoted trainees’ awareness regarding the importance of program fidelity. The goals of our training went beyond a typical program that targets only specific skills and concepts that are necessary to deliver a single intervention. We used the Logic Model as a systematic means to incorporate research and quality improvement in our interventions, which helped trainees to understand the process of “evidence-generating” and accomplished the mission of integrating clinical practice and research. We have previously attempted to teach some basic development and evaluation skills in our programs, but did so in a brief 1 h session, which elicited feedback that there was a need for more of this kind of training. The current workshop used a 3-h session to introduce the Logic Model instead of a 1-h session on program design and assessment in the EFWB TTT (27). We delivered detailed information on the Logic Model and included a group exercise and discussion about the application of the model. Such additional activities provided the trainees with more opportunity to develop confidence and skills to integrate the model into their individual practices.

Our program had several strengths. Our 2-day workshop was short, compared with most reports of TTTs in the literature, which ranged from 3 days to 10 days (24, 40–42). Brevity should make the training less costly and more widely accessible. We used experiential learning methods that have been shown to be effective in enhancing the professional development of social service workers (26, 43). We also provided post-training support by distributing training manuals, which helped promote trainees’ self-efficacy for designing and implementing future interventions (23). Finally, many TTTs in the literature have reported either qualitative evaluations such as narrative feedback and process evaluation (43, 44) or quantitative evaluations (24, 45), but not both. We used the Triangulation Research Method, which includes both quantitative and qualitative assessments to examine the effectiveness of the TTTs, and thus increased the validity of the results.

We acknowledge some limitations in our study. As validated questionnaires were not (and still are not) available in the literature, we used study-specific questionnaires for our targeted outcomes. We assessed only trainees’ perception of knowledge, but did not test their actual knowledge. The attrition rate was high, because our collaborating service organizations had a high staff turnover over the course of the 1-year study resulting in loss of follow-up in some of our trainees. We mitigated this concern somewhat by using the conservative intention-to-treat analysis. Our sample size was small, and the trainees who joined our study might not be representative of their colleagues within and beyond their own service organizations. We had no control group and could not assess the possibility that social desirability bias might have led to over-estimates of effect size. However, we did observe varying effect sizes for different outcomes, some of which diminished at 1-year follow-up, suggesting that primary social desirability effects would not be substantial. A study design with a control group for a TTT may provide stronger evidence, but is less practical and acceptable in community settings.

The need to increase capacity in the community has been well acknowledged. Our study offers a practical example of academic-community partnerships to transfer theory to practice for in-service training. We have provided an operational framework and process to educate frontline social service workers to design, implement, and evaluate their community-based interventions.

## Ethics Statement

Ethical approval was granted by the Institutional Review Board (IRB) of The University of Hong Kong/Hospital Authority Hong Kong West Cluster (HKW IRB reference number: UW12-502) and was retrospectively registered at the National Institutes of Health (Identifier number: NCT01796275). Written informed consents were obtained from the trainees.

## Author Contributions

THL and SC conceived and designed the experiments. MM, AW, and CY contributed the tools and performed the experiments. AL performed the analysis. AL, SC, and T-hL wrote the manuscript. All authors approved the final version of the manuscript.

## Conflict of Interest Statement

The authors declare that the research was conducted in the absence of any commercial or financial relationships that could be construed as a potential conflict of interest.
