# Supplementary material for: An Evaluation of a Train-the-Trainer Workshop for Social Service Workers to Develop Community-Based Family Interventions
Source: Front Public Health. 2017 Jun 30;5:141. doi: 10.3389/fpubh.2017.00141 (PMC5491537; doi:10.3389/fpubh.2017.00141)
Supplement: Supplementary file 1 [file Table_1.PDF]

Supplementary Table 1   The behavioural indicators of the five positive psychology themes

Themes	Behavioural indicators
Joy	- Share happy experiences with family<br>- Wait for everyone to be seated before starting to eat, and say “Let’s eat together!” towards each other<br>- Recall one experience that makes you or your family happy every day
Praise and Gratitude	- Praise the strength and integrity of your family members<br>- Express gratitude to family members by words<br>- Express gratitude to family members by actions, e.g. massage, serving tea
Flow	- Cook/ prepare/ clear/ wash dishes, etc. together with family members<br>- Look for family member’s character strengths, and tell them what you has observed<br>- Focus on cooking/ dining with family, without doing anything else
Savoring	- Slow down the pace of eating<br>- Savour food by observation, focus on its ‘colour’, ‘smell’ and ‘taste’<br>- Treasure good time when dine with family, e.g. stay with family on table though finish dining
Listening	- Listen actively to what family member says, his/her sharing or experiences without intervention<br>- Observe attentively the facial expressions, body languages or other non-verbal expressions of family members<br>- Show understanding towards the feelings/ thoughts/needs of family members
