# Supplementary material for: An Evaluation of a Train-the-Trainer Workshop for Social Service Workers to Develop Community-Based Family Interventions
Source: Front Public Health. 2017 Jun 30;5:141. doi: 10.3389/fpubh.2017.00141 (PMC5491537; doi:10.3389/fpubh.2017.00141)
Supplement: Supplementary file 2 [file Table_2.PDF]

Supplementary Table 2 Demographic characteristic of all trainees, those who completed the one-year follow-up and those who participated in the focus group interviews, compared with those did not

	One-year follow-up			Focus group interviews		
	**Completed (n = 31) Number (%)**	**Did not complete (n = 25) Number (%)**	**p value**	**Participated (n = 13) Number (%)**	**Did not participate (n = 43) Number (%)**	**p value**
Age group, years						
18-24	1 (3)	3 (12)	0.17	2 (15)	2 (5)	0.46
25-34	17 (55)	17 (68)		8 (62)	26 (60)	
35-44	10 (32)	5 (20)		2 (15)	13 (30)	
>=45	3 (10)	0 (0)		1 (8)	2 (5)	
Female	24 (77)	20 (80)	0.82	9 (69)	35 (81)	0.35
Tertiary degree or above	22 (71)	19 (76)	0.67	9 (69)	32 (74)	0.71
Occupation						
Registered social worker	25 (81)	16 (64)	0.36	13 (100)	28 (65)	<0.05*
Service worker	5 (16)	8 (32)		0 (0)	13 (30)	
Teacher	1 (3)	1 (4)		0 (0)	2 (5)	
Social service experience						
Less than 5 years	7 (24)	12 (48)	0.07	6 (46)	14 (32)	0.10
5-9 years	12 (38)	6 (24)		1 (8)	17 (40)	
⩾ 10 years	12 (38)	7 (28)		6 (46)	12 (28)	
Service targets						
Family	22 (71)	12 (48)	0.08	6 (46)	28 (65)	0.22
Children	13 (42)	15 (60)	0.18	7 (54)	21 (49)	0.75
Teenagers	12 (39)	11 (44)	0.69	6 (46)	17 (40)	0.67

Chi-square test was used to compare the difference between two groups, * p value <0.05
