# Supplementary material for: An Evaluation of a Train-the-Trainer Workshop for Social Service Workers to Develop Community-Based Family Interventions
Source: Front Public Health. 2017 Jun 30;5:141. doi: 10.3389/fpubh.2017.00141 (PMC5491537; doi:10.3389/fpubh.2017.00141)
Supplement: Supplementary file 3 [file Table_3.PDF]

Supplementary Table 3 Trainees' knowledge, self-efficacy, attitude and intention in relation to apply the concepts of positive psychology and the Logic Model in interventions over time: Per-protocol analysis (n=31)

	Pre-training	Immediately following training	Six months	One year	Difference between Pre-training and immediately following training	Pre-training and six months	Pre-training and one year
**Positive Psychology**	**Mean ± standard deviation**				**Cohen's d[a]/ p-value**		
Perceived knowledge of the general concept of positive psychology#	3.3 ± 1.1	4.8 ± 0.7	4.6 ± 0.7	4.8 ± 0.5	1.18/ <0.001***	1.00 / <0.001***	1.15/ <0.001***
Self-efficacy in relation to using positive psychology constructs to design interventions#	2.9 ± 0.9	4.4 ± 0.8	4.5 ± 0.7	4.6 ± 0.5	1.36 / <0.001***	1.48 / <0.001***	1.60 / <0.001***
Attitude towards the practice of positive psychology#	4.3 ± 1.0	5.0 ± 0.6	5.0 ± 0.6	5.0 ± 0.6	0.64 / <0.01**	0.64 / <0.01**	0.55 / <0.01**
Intention to apply positive psychology in interventions#	4.8 ± 0.9	5.2 ± 0.7	4.8 ± 0.6	4.8 ± 0.7	0.49 / <0.05*	0.07 / 0.71	0.03 / 0.86
**The Logic Model**							
Perceived knowledge of the general concept of the Logic Model #	3.0 ± 1.0	4.5 ± 0.8	4.5 ± 0.7	4.2 ± 0.7	1.10 / <0.001***	1.30 / <0.001***	0.96 / <0.001***
Self-efficacy in relation to using the Logic Model to design interventions #	3.7 ± 0.8	4.4 ± 0.8	4.5 ± 0.6	4.1 ± 0.6	0.81 / <0.001***	0.84 / <0.001***	0.41 / <0.05*
Attitude towards the practice of the Logic Model #	3.6 ± 1.0	4.4 ± 0.8	4.5 ± 0.6	4.2 ± 0.5	0.71 / <0.001***	0.80 / <0.001***	0.50 / <0.05*
Intention to apply the Logic Model in interventions #	3.7 ± 0.9	4.5 ± 0.8	4.4 ± 0.6	4.0 ± 0.7	0.81 / <0.001***	0.75 / <0.01**	0.30 / 0.11

Per protocol analysis: Including only those who completed all assessments.
Number of questions on positive psychology: perceived knowledge (2 items), self –efficacy (2 items), attitude towards the practice (3 items), intention to apply in practice (1 item)
Number of questions on the Logic Model: perceived knowledge (1 item), self –efficacy (1 item), attitude towards the practice (3 items), intention to apply in practice (1 item)
6-point Likert scale: 1 = strongly disagree; 2 = disagree; 3 = slight disagree; 4 = slight agree; 5 = agree; 6 = strongly agree
Repeated measures analysis of variance and paired T–test comparing the mean at four time points and between two time points, respectively
Difference at four time points: # p value < 0.001
Difference between two time points: *** p value < 0.001, **p value <0.01, * p value < 0.05
[a] Effect size (Cohen's d): small = 0.20, medium = 0.50 and large = 0.80
